# Determination of Polycyclic Aromatic Hydrocarbons and Organic Molecular Tracer Compounds in Dusts Samples from Schools in Puchuncaví and Quintero (Chile)

**DOI:** 10.3390/molecules31050818

**Published:** 2026-02-28

**Authors:** Sonnia Parra, Manuel A. Bravo, Barend L. Van Drooge

**Affiliations:** 1 Laboratorio de Química Analítica y Ambiental, Pontificia Universidad Católica de Valparaíso, Avenida Brasil 2950, Valparaíso 2340025, Chile; 2 Department of Environmental Chemistry, Institute of Environmental Assessment and Water Research (IDAEA-CSIC), c/Jordi Girona 18-26, 08034 Barcelona, Spain

**Keywords:** air pollution, organic molecular tracer compounds, polycyclic aromatic hydrocarbons, primary school

## Abstract

This investigation was conducted in order to gain a first knowledge of concentrations, distribution patterns, and potential sources of 16 US EPA priority polycyclic aromatic hydrocarbons (PAHs) and organic molecular tracer compounds in deposition dust samples collected in the Valparaiso region, Chile. Dust was sampled in schools (indoor and outdoor) that are located in Puchuncaví and Quintero. Source apportionment analysis using the concentrations of PAHs; glucose, mannitol, sucrose, fructose; di-2-ethylhexyl phthalate; hopanes, and levoglucosan as molecular tracer compounds showed three sources of contribution. The first (46.38%) was related to incomplete combustion processes (Acy, Flu, Ant, Flt, Pyr, and BaA), a second source (20%) represented soil+ biomass burning (levoglucosan, α glucose, β glucose, mannitol, sucrose, and fructose), and a third source (10.26%) was dominated only by 27_norhopane, 27_hopane, which are related to traffic. To assess potential health risks for schoolchildren, the study calculated the benzo[a]pyrene equivalent (BaPE) toxicity and the incremental lifetime cancer risk (ILCR). Toxicity equivalent (TEQ) results showed that the main contributor to overall toxicity in PAHs, especially in schools located in Puchuncaví, was benzo[a]pyrene (BaP), followed by benzo[α]anthracene (BaA), benzo[b]fluoranthene (BbF), benzo[k]fluoranthene (BkF), indeno[1,2,3-cd] pyrene (IcdP), and dibenzo[a,h]anthracene (DahA). According to the calculated ILCR values, the highest cancer risk was associated with dust ingestion (both indoor and outdoor) for ∑16PAHs, ranging from 1.14 × 10^−3^ to 8.88 × 10^−4^. This was followed by dermal contact (1.27 × 10^−5^ to 7.27 × 10^−7^) and inhalation (1.22 × 10^−8^ to 9.99 × 10^−9^).

## 1. Introduction

Polycyclic aromatic hydrocarbons (PAHs) are priority contaminants (16 EPA PAHs; USEPA, 2011) due to their adverse effects on human health, which are related to their cytotoxicity, mutagenic properties, and carcinogenicity. Consequently, they are among the most extensively studied organic compounds in environmental research [1,2,3,4,5,6]. As ubiquitous anthropogenic pollutants, PAHs have been identified worldwide in various environmental matrices, including soil [7,8,9], street dust and air [10,11,12], sediments [13,14], water [15], and food [16].

PAHs are semi-volatile organic compounds (SVOCs) that readily bind to particulate matter and dust in indoor environments, while outdoors, they can undergo long-range atmospheric transport [5]. When released into the atmosphere—primarily through human activities—PAHs originate from sources such as fossil fuel-powered vehicles, industrial facilities, and domestic and residential burning processes [17]. Through both dry and wet deposition, a significant fraction of atmospheric PAHs accumulates in soil [18]. Outdoor air pollution and contaminated soil dust can also affect indoor environments [19,20], including schools, through the introduction of road dust via footwear and through infiltration during cross-ventilation [17,21,22]. Dust can act as an important source of indoor pollution for schoolchildren through dermal contact, ingestion, and inhalation following resuspension, allowing contaminants to enter the human body and adversely affect health [23,24,25,26,27]. Moreover, schoolchildren spend several hours per day indoors. Elementary school students are more vulnerable than adults to exposure to particulate matter (fine dust) due to their early developmental stage. Children inhale air at rates approximately 3–5 times higher than adults and are therefore exposed to higher levels of pollution per unit lung area [28,29].

Besides PAHs, numerous other SVOCs are ubiquitous in indoor environments and are frequently detected in indoor air and settled dust [30]. Among these SVOCs, phthalate esters are commonly found in a wide range of building materials and consumer products, including flooring, carpet padding, wall coverings, tiles, furniture, and electronic devices [31,32]. According to European Union risk assessments conducted in 2005, di-(2-ethylhexyl) phthalate (DEHP), among other phthalate esters, was classified as a hazardous substance [33].

The aim of the present study was to determine the concentrations of various polycyclic aromatic hydrocarbons (PAHs) and organic molecular tracer compounds in dust samples collected from schools in Puchuncaví and Quintero (Chile), and to assess the risks associated with schoolchildren’s exposure to these pollutants. In this study, tracer compounds, i.e., hopanes, levoglucosan, and phthalate esters, were used for source apportionment of PAHs derived from fossil fuel combustion emissions, traffic and industrial complexes, biomass burning, and plastics, respectively [17]. To the best of our knowledge, this is the first study to measure hazardous organic pollutants in dust from schools in the Puchuncaví–Quintero area of Chile.

## 2. Materials and Methods

### 2.1. Dust Sampling in the Studied Area

**Figure 1:**
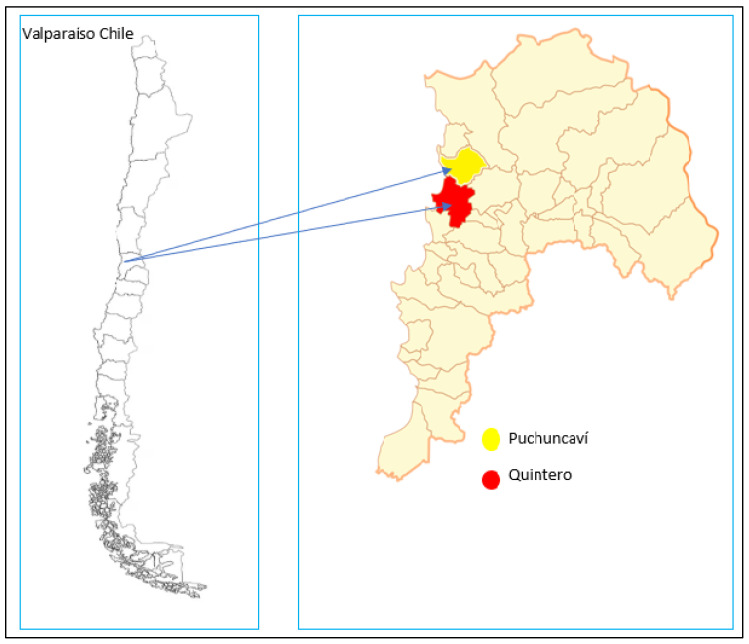
Map of the study area.

The dust samples (n = 33) were collected in eight different schools (indoors and outdoors) in Quintero and Puchuncaví (Chile) during the summer (15 March 2019) and winter (30 August 2019) seasons (Table 1, Figure 1). Samples were collected on aluminum foil with a soft brush. After collection, the samples were stored in polyethylene bags, sealed, and transported back to the laboratory. For the analysis, the samples were dried at room temperature for 72 h and then sieved through a 63 µm nylon sieve to remove largest particles.

### 2.2. Study Area

This study was conducted in the cities of Puchuncaví and Quintero (Chile) (Figure 1), which are situated 155 km northwest of Santiago, in the coastal area of Central (from V region) (Meteorological data study area Table S2). The climate is Mediterranean, with intense winter storms (up to 100–120 mm/d). The study area has been documented to be exposed to environmental pollution from industrial complexes, where emissions of trace elements might include contaminants that pose human health risks.

The most environmentally relevant factories in this area are the CODELCO Division Ventanas copper refinery complex and the AES Gener coal-fired power plant complex. There is a high amount of vehicular traffic in both cities, and their residents use the main access routes to schools daily [34].

As mentioned in the study conducted by Parra et al., 2024 [34], based on the study objectives, we selected schools located at varying distances from the main industrial areas in Puchuncaví and Quintero (Table S3), where weekday vehicular activity related to school operations was higher than in other locations.

### 2.3. Sample Collections

The dust samples (n = 33) were collected in eight different schools (indoors and outdoors) in Quintero and Puchuncaví (Chile) during the summer (15 March 2019) and winter (30 August 2019) seasons (Table 1, Figure 1). Samples were collected following the procedure described by Parra et al., 2024 [34]. After collection, the samples were stored in polyethylene bags, sealed, and transported back to the laboratory. For the analysis, the samples were dried at room temperature for 72 h, sieved through a 63 µm mesh, and stored at ambient temperature before preparation and analysis.

### 2.4. Chemical Analysis

Two grams of dust (≤63 µm) were used for organic analysis following a methodology described elsewhere Van Droogue et al., 2018 [35]. The samples were spiked with deuterated standards (PAH Mix-9 (LGC) and levoglucosan-D_7_ (CIL)). The dust was then extracted using an ultrasonic bath with a dichloromethane/methanol mixture (1:1, *v*/*v*; 3 × 15 mL). After each extraction, the extracts were filtered through a glass syringe (Fortuna Optima) onto glass fiber filters placed in a stainless-steel filter holder (Sartorius, Göttingen, Germany) to remove any remaining particles. To derivatize hydroxyl groups into trimethylsilyl ethers, a 25 µL aliquot was evaporated under a gentle N_2_ stream, followed by the addition of 25 µL of bis(trimethylsilyl) trifluoroacetamide (BSTFA) and 10 µL of pyridine (Merck, Rahway, NJ, USA).

Twenty-five μL of 1-phenyldodecane in isooctane (30 ng) were added and analyzed by gas-chromatography coupled to mass-spectrometry (GC–MS) for the determination of polar compounds, such as saccharides and acids, as well as the phthalate ester di-2-ethylhexyl phthalate.

The remaining extract was hydrolyzed overnight with 5 mL of 6% (*w*/*w*) KOH in methanol. The neutral fraction was recovered with *n*-hexane (3 × 10 mL), vacuum-evaporated to near dryness, and fractionated using a column containing 2 g of alumina, which was eluted with *n*-hexane/dichloromethane (1:2, *v*/*v*). The eluate was concentrated under nitrogen to near dryness, and the combined fractions were vacuum-evaporated to a final volume of 500 µL. Prior to instrumental analysis, the 1-phenyldodecane standard was added.

The sample extracts were injected into a GC–MS (5975; Agilent Technology, Santa Clara, CA, USA) equipped either with a 60 m-fused capillary column HP-5MS 0.25 mm × 25 μm film thickness (Agilent) for the analysis of saccharides and acids, and di-2-ethylhexyl phthalate, and a 30 m column for the analysis of the PAH and hopanes. The oven temperature program started at 60 °C in the first case and 90 °C in the second (holding time 1 min). Then, both programs were heated to 120 °C at 12 °C/min and to 320 °C at 4 °C/min with a final holding time of 10 min. The injector, ion source, quadrupole, and transfer line temperatures were 280 °C, 200 °C, 150 °C, and 280 °C, respectively. Helium was used as carrier gas at 0.9 mL/s. The MS operated in electron impact mode (70 eV). For the purpose of analyzing molecular organic tracers, the quadrupole was run in full scan mode (*m*/*z* 50–650) and in SIM-mode for hopanes and PAH.

Levoglucosan (LEV; biomass burning) and saccharides, as well as di-2-ethylhexyl phthalate (DEHP; plastics), were identified with ions *m*/*z* 204, 204, and 149, respectively, and retention times. Quantification was performed with external standard calibration curves.

The recoveries of the field blank levels and the surrogate standard levoglucosan-d7 (*m*/*z* 206) were used to adjust the concentrations. Benzo[α]anthracene (BAA *m*/*z* 228), chrysene+triphenylene (CHR *m*/*z* 228), benzo [b+j+k] fluoranthene (BFL *m*/*z* 252), benzo[e]pyrene (BEP *m*/*z* 252), benzo[a]pyrene (BAP *m*/*z* 252), indeno[1,2,3-cd] pyrene (IP *m*/*z* 276), and benzo[ghi] perylene (BGP *m*/*z* 276) were among the ion fragment grams used to identify PAHs and hopanes. Moreover, 17(H)α-21(H)β-29-norhopane (norhopane) and 17(H)α-21(H)β-hopane (hopane) were identified in the *m*/*z* 191 mass fragment gram and the corresponding retention times [35].

Quantification was also performed by the external standard method. With the exception of benzo[e]pyrene and the hopanes, which were adjusted using benzo[a] pyrene-d12, the computed concentrations were adjusted for surrogate recoveries, which were made up of deuterated compounds for each distinct PAH. These surrogate standard recoveries exceeded 70% in each case.

The range of field blank levels relative to sample levels was between 1% and 10%. In the standard calibration curves, the lowest measured values were used to calculate the limits of quantification (LOQs). For organic molecular tracers, the LOQ was 0.1 ng g^−1^, whereas for PAHs and hopanes, it was 1.0 ng g^−1^. The corresponding limits of detection (LOD) were 0.011 ng g^−1^ for molecular tracers and 0.1 ng g^−1^ for PAHs and hopanes, respectively.

### 2.5. Statistical Analysis

Principal Component Analysis using PAST software (v5.2.2) was applied on the database, and the resolved components were described by their compound profiles (loadings) and their contribution in each sample (scores).

### 2.6. Health Risk Assessments

For the carcinogenic risk assessment of PAHs in indoor dust from schools in Quintero and Puchuncaví, two indicators were used: BaPE as the total toxicity equivalence (TEQ) and the incremental lifetime cancer risk (ILCR).

#### 2.6.1. BaPE as TEQ

Considering that BaP is the most carcinogenic PAHs, BaP equivalent was used to evaluate the toxicity of PAHs. The BaPE factor was calculated using the next equation [36,37].

(1) BaPE(TEQ)=∑Cn×TEF

where TEF (ng/g) is the toxic equivalence factor of that PHAs [38] and Cn is the average concentration (ng/g) of the PAH in the indoor dust of schools.

#### 2.6.2. Incremental Lifetime Cancer Risk (ILCR)

To evaluate the cancer risk among students in school from Puchuncaví and Quintero, the ILRC model was applied to determinate human health risk from the dust bound PAHs via three main pathways i.e., ingestion, inhalation, and dermal contact [39,40].

(2) $${ILCRs}_{inhalation}=\frac{\left(C\times CSF\left(inh\right)\times \sqrt[{3}]{\left(\frac{BW}{70}\right)}\times IR\left(inh\right)\times EF\times ED\right)}{BW\times AT\times PEF}$$

(3) $${ILCRs}_{Dermal}=\frac{\left(C\times CSF\left(der\right)\times \sqrt[{3}]{\left(\frac{BW}{70}\right)}\times SA\times AF\times ABS\times ED\right)}{BW\times AT\times 10^{6}}$$

(4) $${ILCRs}_{Ingestion}=\frac{\left(C\times CSF\left(ing\right)\times \sqrt[{3}]{\left(\frac{BW}{70}\right)}\times {IR}_{ing}\times EF\times ED\right)}{BW\times AT\times 10^{6}}$$

where C (μg/g) is the total PAH concentration, PEF is the particle emission factor (1.36 × 10^9^ m^3^/kg), ABS is the dermal adsorption fraction (0.13 both adult and children), AF is the dermal adherence factor (0.07 mg/cm^2^/h for adult and 0.2 mg/cm^2^/h for children), SA is the dermal surface exposure (5700 cm^2^ for adult and 2800 cm^2^ for children), IR_ing_ is the dust intake rate (100 mg/day for adult and 200 mg/day), IR_inh_ is the inhalation rate (20 m^3^/day for adult and 7.6 m^3^/day for children), ED is the exposure duration (24 years for adult and 6 years children), EF is the exposure frequency (180 days/year), AT is the average life span (25,550), and BW is body weight taken as 70 kg for adult, as well as 15 kg for children. The CSFing, CSFinh, and CSFder values were taken as 7.3, 3.85, and 25, respectively [26,38,41,42].

## 3. Results and Discussion

### 3.1. Organic Tracer Compound Concentrations

Primary saccharides have been used as tracers for organic soil dust as they are tracer compounds for vegetal debris and fungi [43,44]. Sucrose and glucose are mainly derived from plant materials [45,46]. Amongst sugar alcohols, mannitol is a tracer of airborne fungi [47,48,49]. In this study, glucose, mannitol, sucrose, and fructose are the compounds found in higher concentrations (ng/g) (see Table 2) and directly related to soil dust in combination with microorganisms. Higher indoor concentrations were possibly affected by sugar-holding food consumption due to high concentrations of sucrose and glucose inside schools. Spearman rank correlation coefficients calculated between pollutant levels in school dust show correlations between statistically significant glucose, mannitol, and sucrose (*p* ˂ 0.001).

In this study, the maximum concentration of DEHP in indoor school dust was 373 µg/g at Santa Filomena School, whereas the maximum outdoor concentration was 473 µg/g at Ingles Quintero School (Table 2). These concentrations are lower than those reported by Kim et al. (2022) [32], who found values up to 1310.00 µg/g but are within the range reported by Blanchard et al. (2014) [30] for settled dust samples. Higher DEHP concentrations observed in some schools are likely associated with the presence of plastic toys in classrooms, the use of plastic-based classroom materials, and PVC flooring, which are recognized as major sources of phthalates in indoor dust [28,50].

Hopanes were used as tracers of mineral oils originating from transportation-related sources, including fossil fuel combustion and residues of unburned lubricating oils [51,52]. In school environments, their presence is likely the result of outdoor air infiltrating indoor spaces through ventilation systems [53]. Among the hopanes analyzed, 7α(H),21β(H)-29-norhopane (Norhop) and 17α(H),21β(H)-hopane (Hop) were the most abundant, and their concentrations were highly correlated (R^2^ = 0.97, *p* < 0.001). During winter, the highest indoor concentrations were recorded at Básica Chocota School (847 ng/g), in agreement with values reported by Van Drooge et al. (2020) [17]. These elevated levels likely reflect the school’s location in a high-traffic area. In contrast, the lowest hopane concentrations were observed in outdoor dust samples from Santa Filomena School (74.8 ng/g).

Notably, coal combustion can release levoglucosan, hopanes, and PAHs [54]; however, due to their strong correlation, hopanes were selected as the primary tracers of traffic-related emissions in this study.

Levoglucosan is a common indicator of burning biomass [55,56]. Levoglucosan has been found in aerosols [17,57,58] and soils [59], according to earlier research. In the current study, indoor dust samples from Greda School showed the greatest amounts (1.207 ng/g) during the winter (Table 2). Levoglucosan levels were around three times greater in indoor samples than in outdoor school settings.

PAHs are common byproducts of incomplete combustion and are considered ubiquitous indoor pollutants owing to their widespread sources, with the possibility after the penetration of outdoor air to the indoor air through ventilation [17,30,35,40,60]. Three-ring low-molecular weight (Acy, Ace, Flu, Phe, Ant) PAHs occur in the atmosphere mostly in the vapor state and are indicative of incomplete combustion processes [26,61,62], while 4-ring PAHs exist between the particulate and gaseous states.

In the schools studied, fossil fuel combustion from motorized vehicles represents the main source of PAHs in urban areas [17]. Concentrations and compositional profiles of PAHs in indoor and outdoor dust samples are summarized in Table 3 and Table S1, and Figure 1.

The most frequent PAH species identified in indoor dust samples collected from schools in Puchuncaví was phenanthrene (Phe; 3-ring), accounting for 17%, followed by pyrene (Pyr; 4-ring) and chrysene (Chr; 4-ring), which represented 14% and 12%, respectively. In indoor dust samples collected from schools in Quintero, the predominant PAHs were Pyr (19%) and fluoranthene (Flt; 4-ring) (13%), followed by Chr (12%).

Similarly, in outdoor dust samples collected from schools in Puchuncaví and Quintero, pyrene (Pyr) was the predominant PAH, accounting for 17% and 16%, respectively, followed by phenanthrene (Phe) (15% and 12%) and chrysene (Chr) (14%).

Therefore, based on the obtained results, PAHs tend to accumulate in indoor dust, indicating that indoor environments may act as important reservoirs of PAH contamination and serving as a relevant indicator of indoor pollution.

This fact could be due to the existence of different industries operating rampantly in the area, leading to generation pollution atmospheric, which agrees with the literature where 3- and 4-ring PAHs are reported to be the main contributor in settled dust [63].

Highest ΣPAH concentrations (summer) were measured inside schools from Puchuncaví, including Greda (Alerces) (556 ng/g), Greda (464 ng/g), and Santa Filomena (352 ng/g), with the latter being located in Quintero. Equally, in winter, the highest concentrations were reported in Greda (Alerces) School (746 ng/g), Santa Filomena (507 ng/g). In the case of outdoor conditions in schools, the highest ΣPAH concentrations were measured in the Greda School (summer) 537 ng/g and (winter) 497 ng/g. Equally, the lowest ΣPAH concentrations were measured in the summer (outdoor) at Santa Filomena school (43 ng/g) and in the Básica la Chocota school (76 ng/g).

In general, the relative composition of individual PAHs was dominated by phenanthrene, pyrene, and chrysene, followed by benzo[ghi]perylene and other PAHs at lower concentrations. In dust samples collected from indoor school environments in Puchuncaví during the summer, higher abundances of benzo[b+j+k] fluoranthene were observed. In contrast, during winter, pyrene exhibited the highest abundance, followed by chrysene and fluoranthene. Similarly, in dust samples collected from indoor schools in Quintero, both in winter and summer, benzo[a]pyrene was the most abundant, followed by chrysene and pyrene (Table 2). Across the complete dataset, these correlations were statistically significant, suggesting that combustion is the primary source of PAHs.

### 3.2. Benzo(a)pyrene Toxicity Equivalence (BaPE as TEQ)

The calculated BaPE data expressed as TEQ are presented in Table 4. Median BaPE values indicated that the primary contributors to overall toxicity in schools located in Puchuncaví were BaP, followed by BaA, BbF, BkF, IcdP, and DahA. Similarly, the overall toxicity in schools located in Quintero was associated with the same PAHs, although their contributions to BaPE as TEQ were considerably lower.

These findings suggest that exposure to these PAHs in both Puchuncaví and Quintero poses a potential carcinogenic risk for schoolchildren. Notably, all schools exhibited the highest BaPE as TEQ levels for BaP (5.6–28.8 ng/g), which may be attributed to traffic emissions and combustion sources in the study areas.

### 3.3. Incremental Lifetime Cancer Risk (ILCR)

Table 5 presents the ILCR values, which estimate the cancer risk associated with human exposure to PAHs from pollution sources via ingestion, dermal contact, and inhalation. The highest cancer risk was observed through dust ingestion (indoor and outdoor) for Σ16PAHs (1.14 × 10^−3^–8.88 × 10^−4^), followed by dermal exposure (1.27 × 10^−5^–7.27 × 10^−7^) and inhalation (1.22 × 10^−8^–9.99 × 10^−9^). When compared with USEPA reference values (ranging from 1 × 10^−6^ to 1 × 10^−4^), the ILCR values for ingestion exceeded the acceptable risk level. In Puchuncaví, the highest cancer risk via dust ingestion for children was observed at Greda School, followed by Chocota and Horcón Schools. In Quintero, all schools exhibited similar ILCR values on the order of 10^−4^.

These results indicate that combustion is likely a major factor influencing the carcinogenic risk of PAHs, a consideration that should be emphasized when assessing exposure via ingestion.

### 3.4. Source Identification

**Figure 2:**
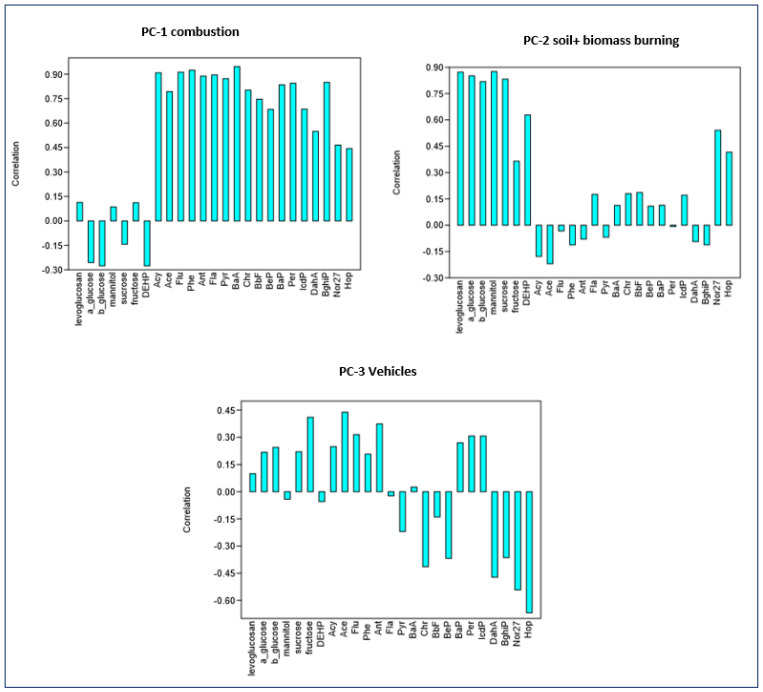
PCA loading% of the analyzed compounds in three components.

The concentrations of the analyzed compounds were evaluated with PCA, and the resolved components are shown in Figure 2. In the present study, loadings were considered significant for values of 0.7 or greater [65].

A three-component solution explains 77% of the variance in the dataset (Figure 2). In this model, the component associated with combustion-related PAHs (CP-1) was most prominent in Greda School (Alerces), which is consistent with the distance to the pollution source (Table S3). In contrast, a component associated with traffic sources (norhopane, hopane, and PAHs; CP-3) was predominant in Greda, Chocota, Ingles Quintero, and Santa Filomena schools, all located in areas with heavy vehicular traffic, where residents frequently use the main routes to access these schools.

Additionally, the contribution of a soil and biomass burning source (saccharides; CP-2) was strongest in Santa Filomena School.

**Figure 3:**
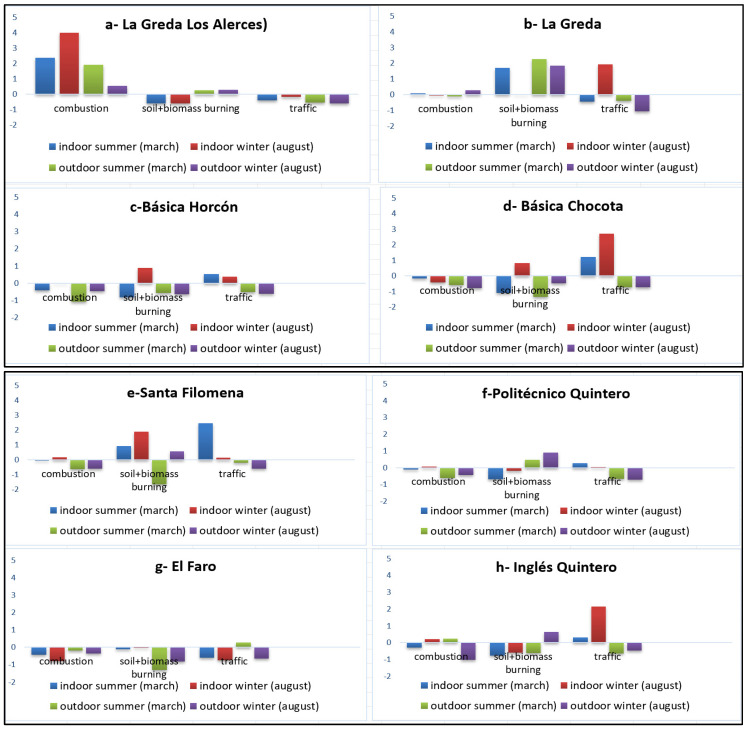
(**a**) PCA score values of the three components in the outdoor and indoor of the schools of Puchuncaví (**a**–**d**). (**b**) PCA score values of the three components in the outdoor and indoor of the schools of Quintero (**e**–**h**).

The schools located in Puchuncaví were influenced by indoor pollution originating from outdoor combustion, traffic, and indoor soil and biomass burning sources, respectively. In contrast, in schools located in Quintero, traffic was the predominant indoor source, followed by soil and biomass burning (Figure 3a,b).

Factor 1 (Figure 2) accounted for 46.38% of the total variance and exhibited strong positive loadings for Acy, Flu, Ant, Flt, Pyr, and BaA. Three-ring PAHs are indicative of incomplete combustion processes [14,38,60], while 4-ring high molecular weight PAHs (Flt, Pyr, and BaA) typically originate from pyrogenic sources such as coal combustion [36,64].

Factor 2 (Figure 2) explained 20% of the variance and showed positive loadings for levoglucosan, α-glucose, β-glucose, mannitol, and sucrose, suggesting a source related to soil and biomass burning.

Factor 3 (Figure 2) contributed 10.26% of the variance and was dominated by 27-norhopane and 27-hopane, which are associated with traffic emissions.

## 4. Conclusions

This study provides the first assessment of organic molecular tracers and polycyclic aromatic hydrocarbons (PAHs) in settled dust from schools in the Puchuncaví–Quintero region, Chile, demonstrating that school dust is a significant source of harmful organic pollutants.

Total Σ16 PAH concentrations in dust ranged from 0.04 to 204 ng/g. The most affected schools were Greda in Puchuncaví and Santa Filomena in Quintero, likely due to their locations in high-traffic areas.

PAH profiles were dominated by low- and medium-molecular-weight compounds (3–4 rings), such as phenanthrene, pyrene, and chrysene, indicating that incomplete combustion is the primary source of contamination. Source apportionment using molecular tracers and multivariate analysis confirmed that traffic emissions and fossil fuel combustion are the main contributors, while biomass burning and soil dust also play a role in certain schools.

DEHP concentrations highlight the influence of indoor sources, including plastic toys, classroom materials, and PVC flooring.

Toxicity assessment (BaPE) revealed that benzo[a]pyrene (BaP), followed by BaA, BbF, BkF, IcdP, and DahA, is the major contributor to carcinogenic risk. Dust ingestion was identified as the primary exposure route, followed by dermal contact and inhalation.

The analysis of molecular tracers, source apportionment, multivariate statistics, and health risk indicators provides a robust interpretation of pollution sources and associated hazards in schools. A clear spatial correlation was observed between emission sources and toxicological impact, with higher PAH loads, elevated hopane concentrations, and increased carcinogenic potential in schools near industrial complexes or high-traffic areas.

## Figures and Tables

**Table 1:** Schools where samples were collected.

School	Place	Latitude S	Longitude W
Greda los Alerces	Puchuncaví	32.736.903	71.460.276
La Greda	Puchuncaví	32.747.420	71.475.076
Básica Chocota	Puchuncaví	32.730.091	71.487.255
Básica Horcón	Puchuncaví	32.712.978	71.488.007
El Faro	Quintero	32.776.909	71.530.828
Santa Filomena	Quintero	32.786.760	71.528.709
Inglés Quintero	Quintero	32.783.022	71.531.031
Politécnico Quintero	Quintero	32.789.093	71.526.921
Reference	Curauma	33.146451	71.569935

**Table 2:** Average concentrations of analyzed organic compounds (ng/g).

	School	Levoglucosan	α Glucose	β Glucose	Mannitol	Sucrose	Fructose	DEHP	Norhopane	Hopane
Seasons	Reference	79	3899	3957	338	9513	95,973	1854	81.64	113.98
summer (indoor)	La Greda (Los Alerces)	295	12,405	12,920	228	52,641	76,725	8920	143.38	156.79
	Santa Filomena	636	484,220	512,210	21,451	831,759	147,975	372,622	303.39	298.02
	Básica Horcón	215	145,801	158,781	1742	1,123,390	73,658	122,626	168.55	166.93
	El Faro	40	9996	10,551	80	472,064	12,971	42,484	86.82	123.60
	La Greda	298	43,157	46,015	517	80,356	52,106	27,445	225.28	305.55
	Politécnico Quintero	407	95,135	107,595	4097	627,116	142,797	208,993	75.84	96.23
	Inglés Quintero	337	147,298	155,576	2448	826,024	165,156	129,893	106.00	132.41
	Básica La Chocota	346	340,428	422,897	788	879,381	253,726	96,784	153.69	168.50
Winter (Indoor)	La Greda (Los Alerces)	224	10,795	12,761	1059	360,082	202,037	8826	170.80	179.45
	Santa Filomena	766	19,317	22,034	12,398	532,435	250,024	117,675	315.26	352.24
	Básica Horcón	453	132,909	133,253	11,408	453,330	99,088	97,817	188.89	211.81
	El Faro	198	13,159	14,898	701	211,087	313,518	117,184	202.43	235.49
	La Greda	1207	353,310	390,340	7963	1,145,896	204,170	286,715	146.75	162.83
	Politécnico Quintero	371	51,960	58,975	2931	574,085	152,543	127,480	136.89	181.62
	Inglés Quintero	619	386,743	407,202	7358	1,570,939	150,194	245,621	157.42	185.24
	Básica La Chocota	898	470,643	429,536	12,902	1,584,915	235,542	315,352	390.54	456.57
Summer (outdoor)	La Greda (Los Alerces)	171	19,809	23,771	304	24,437	161,546	10,266	224.25	263.02
	Santa Filomena	74	175,624	211,877	216	10,557	17,148	5953	35.58	39.17
	Básica Horcón	92	2022	2253	176	4853	124,763	364,474	120.30	155.45
	El Faro	213	302,945	339,534	1241	94,665	99,247	20,174	48.06	69.02
	La Greda	197	22,731	24,318	349	35,169	27,362	17,195	346.63	470.68
	Politécnico Quintero	117	27,182	29,502	173	75,316	44,238	64,024	195.94	252.78
	Inglés Quintero	296	23,636	27,156	955	44,337	478,322	83,299	103.28	113.88
	Básica La Chocota	72	12,451	13,527	125	102,611	156,108	14,568	56.74	69.55
winter (outdoor)	La Greda (Los Alerces)	264	25,647	31,232	488	210,752	131,198	13,829	174.30	210.29
	Santa Filomena	179	1124	4599	82	119,649	7062	1473	221.02	300.10
	Básica Horcón	117	16,317	17,223	1014	127,409	101,170	107,967	97.25	126.63
	El Faro	315	1650	5191	140	25,108	45,435	1944	78.28	107.30
	La Greda	179	2988	3187	430	98,899	73,534	28,230	224.90	278.92
	Politécnico Quintero	75	5422	5488	81	43,113	26,555	28,071	153.03	242.78
	Inglés Quintero	110	9479	10,349	328	73,072	68,810	472,374	221.35	257.44
	Básica La Chocota	85	5474	5638	49	30,389	23,273	61,461	130.19	166.35

**Table 3:** Concentrations of polycyclic aromatic hydrocarbons (PAHs) (ng g^−1^) in dust in schools.

	Indoor			Outdoor		
PAHs	Average	Min	Max	Average	Min	Max
Acy	0.79	0.04	2.60	0.66	0.09	1.87
Ace	1.93	0.34	9.40	1.55	0.69	5.82
Flu	1.95	0.76	7.61	1.44	0.31	5.15
Phe	43.80	10.28	204.37	33.81	5.05	106.52
Ant	4.64	1.23	17.93	3.82	0.53	12.81
Flt	36.50	7.15	84.92	24.01	4.31	55.12
Pyr	49.13	7.37	99.16	41.55	7.50	97.31
BaA	14.63	2.55	40.20	11.06	1.16	29.02
Chr	37.15	8.54	72.95	34.74	6.11	105.79
BbF	33.95	7.31	65.60	23.48	4.64	52.47
Bep	23.38	4.90	43.36	21.22	3.75	42.75
BaP	13.57	2.94	31.64	9.17	1.40	21.94
PE	4.49	1.14	19.55	3.33	0.68	11.71
IcdP	11.80	3.50	24.35	8.20	1.99	20.96
dBahA	3.66	0.52	8.80	3.94	0.41	13.97
Bghip	22.77	6.93	50.27	25.24	3.60	57.08
ƩPAHs	304.2			247.21		

**Table 4:** BaPE as TQE profile of analyzed PAHs in indoor dust of schools from Puchuncaví and Quintero.

Analytes	Toxic Equivalent Factors (TEF) [64]	BaPE as TQE Exposure Profile (ng/g)								
		Puchuncaví					Quintero			
		Reference	La Greda (Alerces)	La Greda	Básica La Chocota	Básica Horcón	El Faro	Santa Filomena	Inglés Quintero	Politécnico Quintero
Phe	0.001	0.02	0.2	0.1	0.02	0.02	0.01	0.1	0.02	0.02
Ant	0.01	0.02	0.2	0.1	0.03	0.02	0.02	0.1	0.03	0.03
FLT	0.001	0.02	0.1	0.0	0.01	0.03	0.03	0.1	0.02	0.03
Pyr	0.001	0.1	0.1	0.1	0.01	0.03	0.1	0.1	0.02	0.03
BaA	0.1	0.4	3.6	1.7	0.9	1.4	0.5	2.0	0.7	1.5
Chr	0.01	0.2	0.6	0.6	0.3	0.3	0.2	0.6	0.2	0.3
BbjkF	0.1	1.3	5.9	6.2	3.3	4.1	2.8	5.7	2.2	3.9
BaP	1	7.5	28.8	13.5	6.7	10.5	5.6	14.7	16.3	15.6
Icdp	0.1	0.3	2.3	1.1	0.6	2.0	0.4	1.6	0.9	1.0
DahA	1	2.1	3.7	5.5	2.4	4.1	5.5	5.2	1.7	1.9
Bghip	0.01	0.3	0.4	0.4	0.1	0.2	0.1	0.3	0.1	0.2

**Table 5:** Cancer risk of PAHs in indoor and outdoor dust of the study schools.

Study Schools	Sampling Location	Season	Children		
			Inhalation	Dermal	Ingestion
La Greda (Los Alerces)	Indoor	summer	2.02 × 10^−8^	9.48 × 10^−6^	1.37 × 10^−3^
		winter	2.71 × 10^−8^	1.27 × 10^−5^	1.84 × 10^−3^
	Outdoor	summer	1.95 × 10^−8^	9.16 × 10^−6^	1.32 × 10^−3^
		winter	1.36 × 10^−8^	6.37 × 10^−6^	9.20 × 10^−4^
La Greda	Indoor	summer	1.68 × 10^−8^	7.92 × 10^−6^	1.14 × 10^−3^
		winter	1.06 × 10^−8^	5.00 × 10^−6^	7.23 × 10^−4^
	Outdoor	summer	1.78 × 10^−8^	8.38 × 10^−6^	1.21 × 10^−3^
		winter	1.80 × 10^−8^	8.47 × 10^−6^	1.22 × 10^−3^
Básica La Chocota	Indoor	summer	4.40 × 10^−9^	2.07 × 10^−6^	2.99 × 10^−4^
		winter	7.44 × 10^−9^	3.50 × 10^−6^	5.05 × 10^−4^
	Outdoor	summer	2.76 × 10^−9^	1.30 × 10^−6^	1.87 × 10^−4^
		winter	4.54 × 10^−9^	2.13 × 10^−6^	3.08 × 10^−4^
Básica Horcón	Indoor	summer	4.29 × 10^−9^	2.02 × 10^−6^	2.91 × 10^−4^
		winter	1.31 × 10^−8^	6.15 × 10^−6^	8.88 × 10^−4^
	Outdoor	summer	3.17 × 10^−9^	1.49 × 10^−6^	2.15 × 10^−4^
		winter	6.04 × 10^−9^	2.84 × 10^−6^	4.10 × 10^−4^
El Faro	Indoor	summer	8.46 × 10^−9^	3.98 × 10^−6^	5.74 × 10^−4^
		winter	6.80 × 10^−9^	3.20 × 10^−6^	4.62 × 10^−4^
	Outdoor	summer	4.63 × 10^−9^	2.18 × 10^−6^	3.15 × 10^−4^
		winter	5.43 × 10^−9^	2.55 × 10^−6^	3.69 × 10^−4^
Santa Filomena	Indoor	summer	1.28 × 10^−8^	5.99 × 10^−6^	8.65 × 10^−4^
		winter	1.84 × 10^−8^	8.65 × 10^−6^	1.25 × 10^−3^
	Outdoor	summer	1.55 × 10^−9^	7.26 × 10^−7^	1.05 × 10^−4^
		winter	8.70 × 10^−9^	4.09 × 10^−6^	5.91 × 10^−4^
Inglés Quintero	Indoor	summer	5.93 × 10^−9^	2.79 × 10^−6^	4.02 × 10^−4^
		winter	7.31 × 10^−9^	3.43 × 10^−6^	4.96 × 10^−4^
	Outdoor	summer	9.99 × 10^−9^	4.70 × 10^−6^	6.78 × 10^−4^
		winter	7.03 × 10^−9^	3.30 × 10^−6^	4.77 × 10^−4^
Politécnico Quintero	Indoor	summer	7.63 × 10^−9^	3.59 × 10^−6^	5.18 × 10^−4^
		winter	9.43 × 10^−9^	4.43 × 10^−6^	6.40 × 10^−4^
	Outdoor	summer	8.54 × 10^−9^	4.02 × 10^−6^	5.80 × 10^−4^
		winter	1.22 × 10^−8^	5.75 × 10^−6^	8.30 × 10^−4^

## Data Availability

The data used to support the findings of this study are available from the corresponding author upon request.

## Supplementary Materials

The following supporting information can be downloaded at: https://www.mdpi.com/article/10.3390/molecules31050818/s1, Table S1: PAHs concentrations (ng g^−1^) in school dust samples; Table S2: Meteorological data of study area; Table S3: Distance of selected schools to the industrial complex located in the study area (The cities of Puchuncaví and Quintero).
