# Supplementary material for: Determination of Polycyclic Aromatic Hydrocarbons and Organic Molecular Tracer Compounds in Dusts Samples from Schools in Puchuncaví and Quintero (Chile)
Source: Molecules. 2026 Feb 28;31(5):818. doi: 10.3390/molecules31050818 (PMC12985584; doi:10.3390/molecules31050818)
Supplement: Supplementary file 1 [file molecules-31-00818-s001.zip › molecules-4063934-supplementary.pdf]

Determination of polycyclic aromatic hydrocarbons and organic molecular tracer compounds in dusts samples from schools in Puchuncavi and Quintero (Chile)

Supplementary content

Sonnia Parra[a,*], Manuel A. Bravo[a], Barend L. Van Drooge[b]

[a] Laboratorio de Química Analítica y Ambiental, Pontificia Universidad Católica de Valparaíso, Avenida Brasil 2950, Valparaíso, Chile.

[b]Institute of Environmental Assessment and Water Research (IDÆA-CSIC), Dept. Environmental Chemistry, c/Jordi Girona 18-26, 08034 Barcelona, Spain

* Corresponding author: Sonnia Parra

E-mail address: sonnia.parra@pucv.cl

Keywords. Air pollution, organic molecular tracer compounds, polycyclic aromatic hydrocarbons, primary school

Table S1. PAHs concentrations (ng g^{-1}) in schooldust samples

			ACY	ACE	FLN	PHE	ANT	FLT	PYR	BaA	CHR	BbF	Bep	BaP	PE	IcdP	dBahA	Bghip	∑PHAs
		Reference	0,84	0,34	1,17	19,88	2,31	19,60	57,35	3,64	15,70	10,98	14,93	7,45	4,52	3,50	2,11	26,78	
		La Greda (Los Alerces)	2,02	5,51	5,17	143,63	13,03	62,03	81,92	30,81	51,58	37,58	29,97	25,87	8,59	21,58	1,29	35,64	556
		Santa Filomena	0,71	1,25	1,83	33,07	2,33	53,63	63,86	16,70	50,24	50,70	20,93	13,03	3,12	13,36	1,64	25,11	352
		Básica Horcón	0,16	1,13	0,81	10,28	2,28	9,52	7,97	3,05	11,21	27,15	9,58	5,64	1,14	19,27	2,13	6,96	118
		El Faro	0,31	2,11	0,82	11,10	1,23	25,34	80,37	2,96	8,54	39,51	33,37	8,30	1,50	4,67	6,25	6,93	233
		La Greda	0,83	1,35	1,94	56,36	4,75	40,64	66,61	18,59	72,95	65,60	41,65	18,72	5,14	11,28	7,62	50,27	464
		Politécnico Quintero	0,66	1,01	0,95	16,60	2,76	25,53	30,47	13,78	28,29	32,62	15,29	14,16	2,92	5,39	1,07	18,91	210
		Inglés Quintero	0,53	1,06	0,89	14,93	2,80	23,84	22,81	7,62	23,51	18,56	12,97	10,07	1,57	7,01	2,84	12,39	163
	summer	Básica La Chocota	0,39	1,79	0,95	11,54	1,84	7,15	7,37	2,55	14,93	37,58	14,87	3,91	2,90	3,81	0,79	8,98	121
		La Greda (Los Alerces)	2,60	9,40	7,61	204,37	17,93	78,32	99,16	40,20	63,02	59,98	35,53	31,64	19,55	24,35	6,18	45,77	746
		Santa Filomena	1,19	1,87	3,41	71,82	6,79	84,92	89,82	22,97	69,17	44,70	25,31	16,37	2,85	19,34	8,80	37,75	507
		Básica Horcón	0,76	1,12	0,92	24,51	2,38	57,88	50,46	25,59	52,14	40,32	30,33	15,33	3,71	20,54	6,03	28,48	360
		El Faro	0,74	1,42	0,76	16,21	2,68	29,75	61,31	7,34	23,79	7,31	4,90	2,94	2,20	3,78	4,69	17,71	188
		La Greda	0,04	0,74	1,96	37,39	4,67	33,35	50,33	15,97	50,09	37,53	17,31	8,21	2,09	11,31	3,42	19,12	294
		Politécnico Quintero	0,80	1,29	1,46	27,56	3,40	30,34	29,66	16,08	36,25	32,06	23,01	17,00	3,44	13,70	2,75	21,16	260
		Inglés Quintero	0,53	0,43	1,14	19,96	3,54	17,08	18,60	5,97	22,79	17,72	43,36	22,60	3,86	10,42	0,52	12,86	201
indoor	winter	Básica La Chocota	0,26	0,93	1,42	25,40	4,26	21,53	17,21	14,86	37,33	17,20	24,22	9,46	7,29	7,29	4,07	12,31	205
		La Greda (Los Alerces)	1,87	5,82	5,15	106,52	12,81	55,12	92,89	29,01	62,13	43,94	28,66	21,94	11,71	13,28	5,53	40,86	537
		Santa Filomena	0,09	1,03	0,36	5,05	0,53	4,31	7,50	1,16	6,11	4,64	3,75	1,40	0,68	1,99	0,41	3,60	43
		Básica Horcón	0,10	0,80	0,31	5,21	1,00	5,92	9,09	3,12	15,26	10,91	11,59	4,73	1,01	4,78	1,73	11,74	87
		El Faro	0,44	1,41	1,05	10,34	1,00	12,88	27,46	4,25	16,89	12,73	10,74	7,37	1,79	5,10	2,40	11,91	128
		La Greda	1,00	2,06	2,01	65,88	5,15	45,80	85,68	29,02	105,79	42,88	42,75	7,44	4,36	8,58	1,78	41,10	491
		Politécnico Quintero	0,76	1,14	0,69	17,52	3,66	26,28	40,35	9,40	42,78	20,61	20,41	8,49	2,46	2,90	3,19	34,87	236
		Inglés Quintero	0,92	1,06	1,28	19,42	8,20	29,17	42,99	13,06	32,71	30,65	23,86	15,89	2,83	20,96	2,70	29,67	275
	summer	Básica La Chocota	0,32	1,27	0,53	5,60	2,62	7,77	8,78	2,77	12,48	8,16	8,20	3,34	1,08	4,07	0,73	8,38	76
outdoor	winter	La Greda (Los Alerces)	1,07	2,59	2,76	64,25	7,44	39,78	63,41	20,85	39,76	35,74	22,54	14,63	6,25	12,52	7,81	32,19	374

		Santa Filomena	0,28	1,32	1,81	48,14	2,32	19,08	34,86	7,57	36,45	19,58	26,27	5,43	3,06	6,32	4,71	22,70	240
		Básica Horcón	0,58	0,93	0,99	17,80	2,61	23,66	25,21	7,96	20,86	17,59	14,12	6,83	1,62	8,16	2,16	15,42	166
		El Faro	0,62	0,81	0,97	14,62	1,91	15,64	19,84	6,01	19,30	15,05	14,66	7,33	1,83	12,69	1,25	17,27	150
		La Greda	1,54	2,07	2,77	70,98	5,66	48,03	97,31	23,30	59,95	35,52	41,41	20,62	5,34	11,07	13,97	57,08	497
		Politécnico Quintero	0,26	0,69	1,11	59,41	3,74	16,20	50,15	8,40	51,16	52,47	37,02	7,33	4,21	6,12	6,50	32,31	337
		Inglés Quintero	0,76	17,00	1,40	19,26	31,49	6,75	21,61	15,21	20,23	10,17	2,82	7,90	5,63	32,20	221,35	257,44	194
		Básica La Chocota	0,55	13,28	1,07	15,29	27,74	4,30	12,64	10,06	13,23	3,71	2,19	4,79	2,46	12,62	130,19	166,35	125

Table S2. Meteorological data of study area

	Quintero	Puchuncaví	Quintero	Puchuncaví
	summer	summer	Winter	Winter
PM_{10}	43	33	39	38
SO_2	7,2	5	6,8	4,2
NO_2	3,7	4,9	7,5	10,6
MH	398	398	247	247
Temperature	14,4	14,4	11,1	11,1
Wind speed	1,7	2,4	2,4	1,9
Precipitation	0	0	0	0

Table S3. Distance of selected schools to the industrial complex located in the study area (The cities of Puchuncaví and Quintero)

School	AES Gener (distance km)	Codelco División Ventanas (distance km)	city
Greda Los Alerces	0.91	1.69	PUCHUNCAVI
La Greda	2.71	3.34	
La Chocota	2.44	3.62	
Bàsica Horcón	4.07	5.27	
El Faro	5.28	5.05	QUINTERO
Santa Filomena	5.38	5.06	
Inglés Quintero	5.67	5.37	
Politécnico Quintero	5.83	5.33	
